# Disquisition on convergence, stability, and data dependence for a new fast iterative process

**DOI:** 10.1038/s41598-024-73261-7

**Published:** 2024-09-30

**Authors:** A. Murali, K. Muthunagai

**Affiliations:** Vellore Institute of Technology, Chennai, India

**Keywords:** Complex valued Banach spaces, M-Fast iterative process, Iterative processes, Stability, Engineering, Mathematics and computing

## Abstract

This paper introduces a novel fast iterative process designed for approximating fixed points of contraction and weak contraction mappings. The study presents strong convergence results for this newly proposed iterative process, and proving its efficiency. Analytical and numerical evidences are provided to establish that the proposed iterative method converges more rapidly than several existing processes. Furthermore, stability results and dependence analysis are presented for the newly developed iterative process, enhancing its practical applicability and robustness.

## Introduction

Fixed point theory is a crucial concept in mathematics and various sciences. It involves expressing problems as equations involving operators and finding solutions by identifying the fixed points of these operators. This theory combines functional analysis, topological theory, and geometry to simplify real-world or theoretical problems into fixed-point problems. Fixed point theory is especially useful in determining whether or not solutions to differential and integral equations exist because these equations govern the behavior of a variety of real-world problems, and the presence of a solution is critical. A fixed point of a mapping is a value that remains unchanged when the mapping is applied to it. In other words, if *F* is a function, a fixed point *x* satisfies $F(x) = x$.

Banach proved a fixed point theorem known as the contraction mapping principle in 1922^1^, which guarantees the presence and uniqueness of a fixed point on a complete metric space. We assume that *D* is a nonempty subset of a Banach space *X*. Let *F* be a self-mapping on *D*. If there exists $\mu \in [0,1)$ such that

$$\begin{aligned} ||Fh_{1}-Fh_{2}|| \le \mu ||h_{1}-h_{2}||,\; \forall h_{1}, h_{2} \in D \subseteq X. \end{aligned}$$

F is called a contraction condition. Numerous articles have been published to enhance the fundamental principle above, leading to its generalization. The majority of these contributions have focused on generalizing the contraction condition within metric spaces. However, once the occurrence of a fixed point for a given mapping is established, determining that fixed point becomes a challenging task. Addressing this challenge effectively involves the utilization of iterative strategies. Therefore, the endeavor to approximate fixed points under diverse contraction conditions is of both theoretical and practical significance. Developing an iterative process with a high convergence rate is crucial for approximating solutions to nonlinear equations. Over the years, many researchers have dedicated their efforts to establishing iterative processes with accelerated convergence rates, specifically within real-valued metric and Banach spaces. We have listed below some one-step iterative processes, namely Picard^2^, Krasnoselskii^3^, and Mann^4^, respectively.

1 $$\begin{aligned} \kappa _1&= \kappa \in D, \kappa _{n+1} = F\kappa _{n}, n\in {\mathbb {N}}. \end{aligned}$$

2 $$\begin{aligned} h_1&= h \in D,h_{n+1} = (1-\gamma )h_{n}+\gamma Fh_{n}, n\in {\mathbb {N}}. \end{aligned}$$

3 $$\begin{aligned} s_1&= s \in D,s_{n+1} = (1-a_n)s_{n}+a_n Fs_{n}, n\in {\mathbb {N}}, \end{aligned}$$

where $\{a_n\}$ & $\gamma$ are in (0, 1). Two-step iterative processes named Ishikawa^5^, S-iterative^6^, Picard Mann hybrid (PMH)^7^, and Picard Krasnoselskii hybrid (PKH)^8^ are detailed below.

4 $$\begin{aligned} q_1&= q \in D, q_{n+1} = (1-a_n)q_{n}+a_n F\kappa _{n}, \kappa _{n} = (1-b_n)q_{n}+b_n Fq_{n}, n\in {\mathbb {N}}. \end{aligned}$$

5 $$\begin{aligned} p_1&= p \in D, p_{n+1} = (1-a_n)Fp_{n}+a_n F\kappa _{n}, \kappa _{n} = (1-b_n)p_{n}+b_n Fp_{n}, n\in {\mathbb {N}}.\end{aligned}$$

6 $$\begin{aligned} u_1&= u \in D, u_{n+1}= F\kappa _{n}, \kappa _{n} = (1-a_n)u_{n}+a_n Fu_{n}, n\in {\mathbb {N}}.\end{aligned}$$

7 $$\begin{aligned} v_1&= v \in D, v_{n+1} = F\kappa _{n}, \kappa _{n} = (1-\gamma )v_{n}+\gamma Fv_{n}, n\in {\mathbb {N}}, \end{aligned}$$

where $\{a_n\}$, $\{b_n\}$ & $\gamma$ are in (0, 1). In 2009, Agarwal et al.^6^ defined the S-iterative method, which approaches faster than Picard, Krasnoselskii, Mann, and Ishikawa iterative methods. In 2013, Khan^7^ defined the PMH iterative process and also proved that the iterative scheme which tends toward faster than all of the Picard, Mann, and Ishikawa processes in the sense of Berinde^9^ for contraction mapping. In^8^, authors proved that the PKH iterative process converges quicker than Picard, Mann, Krasnoselskii, and Ishikawa iterative methods. Next, we give the following three-step iterative process, namely the Picard-Ishikawa hybrid (PIH) whcih is defined in^10^.

8 $$\begin{aligned} {\left\{ \begin{array}{ll} \omega _1 = \omega \in D,\\ \omega _{n+1} = Fv_{n},\\ v_{n} = (1-a_n)\omega _{n}+a_n Fu_{n},\\ u_{n} = (1-b_n)\omega _{n}+b_n F\omega _{n}, n\in {\mathbb {N}}, \end{array}\right. } \end{aligned}$$

where $\{a_n\}$ & $\{b_n\}$ are in (0, 1). In^10^, the author proved that his iterative method converges faster than widely recognized methods such as Picard, Mann, Ishikawa, Krasnoselskii, Picard Mann hybrid, and Picard Krasnoselskii hybrid iterations, as per the criteria outlined by Berinde^9^. Likewise, in^11^ Faik Gursoy et al. introduced the three-step iterative process named Picard-S hybrid (PSH) method that converges faster than the other iteration methods in the literature existing. In^12^, Julee Srivastava used the three-step iterative process namely Picard-S hybrid (PSH), establishing its quicker convergence compared to various other iterative methods like Picard, Mann, Krasnoselskii, Ishikawa, S-iterate, PMH, PKH, and PIH for contraction conditions on real-valued normed linear spaces, for $\{x_n\}$ on real-valued normed linear space, and it is

9 $$\begin{aligned} {\left\{ \begin{array}{ll} x_1 = x \in D, \\ x_{n+1} = Fz_n, \\ z_n = (1-a_n)Fx_{n}+a_n F\kappa _{n} ,\\ \kappa _{n} = (1-b_n)x_{n}+b_n Fx_{n}, \end{array}\right. } \end{aligned}$$

where $$\{a_n\}$$ & $$\{b_n\}$$ are sequences of real numbers in (0, 1). Austine Efut Ofem et al. introduced the three steps $$A^{**}$$ iteration method in^13^, which is a more efficient method for approximating the fixed points of almost contraction mappings and generalized $$\alpha$$-nonexpansive mappings. For another type of three steps iteration method, see^14^. The authors of^15^ provided the AH iterative scheme, a four-step iterative scheme for approximating fixed points of contractive-like mappings, and Reich-Suzuki-type nonexpansive mappings. For additional information regarding four-step iterative schemes, please refer to^16,17^.

Wasfi Shatanawi et al.^18^ introduced the four-step iterative process, namely $SBT_n$, and proved numerically that the iterative process converges faster than Sintunavarat et al.^19^, Agarwal et al., Mann, and Ishikawa iterative processes.

10 $$\begin{aligned} {\left\{ \begin{array}{ll} y_1 = y \in D, \\ y_{n+1} = (1-d_n)Fx_{n}+d_n Fz_{n}, \\ z_n = (1-a_n)Fx_{n}+a_n F\kappa _{n}, \\ x_n = (1-c_n)y_{n}+c_n \kappa _{n}, \\ \kappa _{n} = (1-b_n)y_{n}+b_n Fy_{n}, \end{array}\right. } \end{aligned}$$

where $\{a_n\}, \{b_n\}$, $\{c_n\}$ and $\{d_n\}$ are sequences of real numbers in $[a, 1-a], [b, 1-b], [c, 1-c], [d, 1-d]$ respectively. Hammad et al.^20^ introduced the four-step iterative process named HR, which converges faster than the $K^{*}$ iterative process^21^, S iterative process, Picard-S iterative process, and Thakur iterative process.

11 $$\begin{aligned} {\left\{ \begin{array}{ll} z_1 = z \in D, \\ z_{n+1} = Fy_{n}, \\ y_n = F((1-c_n)x_{n}+c_n F(x_{n})), \\ x_n = F((1-b_n)\kappa _{n}+b_n F(\kappa _{n})), \\ \kappa _{n} = (1-a_n)z_{n}+a_n Fz_{n}, \end{array}\right. } \end{aligned}$$

where $$\{a_n\}, \{b_n\}$$ and $$\{c_n\}$$$$\subset$$ [0, 1]. Recently, Hammad et al.^22^ introduced another four-step iterative process named $$HR^{*}$$ and proved analytically that their iterative processes converge faster than existing iterative processes named JK in^23^.

12 $$\begin{aligned} {\left\{ \begin{array}{ll} o_1 = o \in D, \\ o_{n+1} = (1-c_n)z_{n}+c_n Fz_{n}, \\ z_n = F(F(x_{n})), \\ x_n = F((1-b_n)\kappa _{n}+b_n F(\kappa _{n})), \\ \kappa _{n} = (1-a_n)o_{n}+a_n Fo_{n}, \end{array}\right. } \end{aligned}$$

where $\{a_n\}, \{b_n\}$ and $\{c_n\}$ are sequences of real numbers in (0, 1). In the diverse landscape of pure and applied sciences, spanning domains such as biology, physics, and computer science, the exploration of metric spaces has emerged as a pivotal focus. In 2011, Azam et al.^24^, defined the concept of complex valued metric spaces. His novel concept has assisted researchers in overcoming the disadvantage of being unable to define rational form in cone metric spaces, and it can be used to create complex valued normed spaces as well as complex valued inner product spaces, both of which provide a wealth of potential research topics. More results on complex valued metric spaces have been discussed in^24,25^. However, a conspicuous gap persists in understanding the approximation of fixed points of nonlinear mappings within real-valued metric spaces and real-valued Banach spaces^26–31^. To bridge this void, Okeke, in^32^, introduced the concept of complex valued Banach spaces. Leveraging the iterative techniques proposed by^8,10^, Okeke successfully approximated the fixed points of contraction conditions within these complex valued Banach spaces. Moreover, the PMH iteration and the PKH iteration have been shown to have the same rate of convergence both analytically and numerically.

Motivated by these authors, we propose a new iterative process to approximate fixed points for contraction and weak contraction conditions on complex-valued Banach spaces. We show that the new iterative process converges faster than other iterative processes named S-iterative, Picard Mann hybrid, Picard Krasnoselskii hybrid iterative, Picard Ishikawa hybrid, Picard-S hybrid, *HR*-iterative, and $HR^{*}$-iterative processes. Also, we prove that the new iterative process is strongly convergent on complex-valued Banach spaces. The obtained results are proven both analytically and numerically with examples and visualized for the speed of convergence using Matlab tools for contraction and weak contraction conditions. We prove a small result for nonexpansive mapping using our new iterative process. We discuss the stability of our novel iterative technique and its impact on data dependence for contraction.

## Lemmas and definitions

The purpose of this section is to provide the reader with certain definitions and lemmas that will ensure that they have a better comprehension of our content and will be beneficial in the subsequent section. Let *A* be a linear space over a field ${\mathbb {K}}$, where ${\mathbb {K}} = {\mathbb {R}}$ or ${\mathbb {K}} = {\mathbb {C}}$.

### Lemma 2.1

^32^ Let (*A*, ||.||) be a complex valued Banach space, and let $\{p_n\}$ be a sequence in *A*. Then $\{p_n\}$ converges to *p* if and only if $\left| {\Vert {p_n-p} \Vert }\right| \rightarrow 0$ as $n \rightarrow \infty$.

### Lemma 2.2

^32^ Let (*A*, ||.||) be a complex valued Banach space and $\{p_n\}$ be a sequence in *A*. Then $\{p_n\}$ is a Cauchy sequence if and only if $\left| {\Vert {p_n-p_{n+m}} \Vert }\right| \rightarrow 0$ as $n \rightarrow \infty$.

### Definition 2.1

^9^ Let $\{\ell _n\}, \{m_n\}$ be two sequences of positive numbers that converge to $\ell$ and *m*, respectively. Suppose that $\exists$ a constant *c* such that $\displaystyle \lim _{n\rightarrow \infty } \dfrac{||\ell _n-\ell ||}{||m_n-m||} = c$. If

1. $c = 0$, then $\{\ell _n\}$ converges to $\ell$ faster than $\{m_n\}$ to *m*.
2. $0<c<\infty$, then $\{\ell _n\}_{n = 0}^{\infty }$ and $\{m_n\}_{n = 0}^{\infty }$ have the same rate of convergence.

For more details on the following, one can refer to^9,33–38^.

### Definition 2.2

Let $F, \overset{\sim }{F}$ be two self operators on $B \subseteq A$. We define $\overset{\sim }{F}$ as an approximate operator of *F* if, for any $a \in B$ and a given fixed $\epsilon > 0$, the condition $||Fa-\overset{\sim }{F}a|| \le \epsilon$ holds.

### Definition 2.3

Let $B \subseteq A$ and *S* be self-mapping on *B*. Assume that $p_1 \in B$ and $p_{n+1} = f(S, p_n)$ defines an iterative process that produces a sequence $\{p_n\} \subset B$ and $\{p_n\}$ converges strongly to $a \in F (S) \ne \emptyset$ where *F*(*S*) is the set of all the fixed points of *S*. Let $\{q_n\}$ be any sequence of bounded in *B*, and choose $\epsilon _n = ||q_{n+1}-f(S, q_{n})||$. Then

1. The iterative process $$\{p_n\}_{n=0}^{\infty }$$ defined by $$p_{n+1} = f(S, p_n)$$ is said to be *S*-stable on *B* if $$\lim _{n\rightarrow \infty } \epsilon _n$$ = 0, $$\implies$$$$\lim _{n\rightarrow \infty } q_n = a$$.
2. The iterative process $$\{p_n\}_{n=0}^{\infty }$$ defined by $$p_{n+1} = f(S, p_n)$$ is said to be at-most *S*-stable on *B* if $$\displaystyle \sum _{n=1}^{\infty } \epsilon _n < \infty$$, $$\implies$$$$\lim _{n\rightarrow \infty } q_n = a$$.

### Lemma 2.3

^9^ Let $$\sigma$$ be a real number in the range $$0 \le \sigma < 1$$, and consider a sequence of positive numbers $$\{\epsilon _n\}_{n=0}^{\infty }$$ such that $$\displaystyle \lim _{n \rightarrow \infty }$$$$\epsilon _n = 0$$. Then, for the sequence of positive numbers $$\{p_n\}_{n=0}^{\infty }$$ satisfying the condition $$p_{n+1} \le \sigma p_n + \epsilon _n$$ for all $$n \ge 0$$, we have $$\lim _{n \rightarrow \infty } p_n = 0.$$

.

### Lemma 2.4

^39^ Let $\{q_n\}$ and $\{\rho _n\}$ be non-negative real sequences satisfying $q_{n+1} \le (1-\mu _n)q_{n} +\rho _{n}$, where $\mu _n \in (0,1), \forall n\ge n_0$, $\displaystyle \sum _{n=1}^{\infty } \mu _n = \infty$ and $\frac{\rho _n}{\mu _n} \rightarrow 0$ as $n \rightarrow \infty$, then $\displaystyle \lim _{n\rightarrow \infty }q_n = 0$.

### Lemma 2.5

^40^ Let $\{q_n\}_{n=0}^{\infty }$ denote a non-negative real sequence that adheres to the inequality:

$$                   q_{n+1} \le (1-\nu _n) q_{n} +\nu _{n}\delta _n$$,

where $$\nu _n \in (0,1) \forall n \in {\mathbb {N}}$$, $$\sum _{n=1}^{\infty } \nu _n = \infty$$, and $$\delta _n \ge 0$$$$\forall$$$$n \in {\mathbb {N}}$$. Given that there exists $$n_0 \in {\mathbb {N}}$$ such that $$n \ge n_0$$, it follows that $$0 \le \displaystyle \limsup _{n\rightarrow \infty }{q_n} \le \displaystyle \limsup _{n\rightarrow \infty }{\delta _n}$$.

Let (*A*, ||.||) be a complex valued Banach space, and *F* be a self-mapping on $$B \subseteq A$$. If $$\exists$$$$\mu \in (0,1)$$ such that

13 $$\begin{aligned} ||Fh_{1}-Fh_{2}|| \preceq \mu ||h_{1}-h_{2}||,\; \forall h_{1}, h_{2} \in B \subseteq A, \end{aligned}$$

*F* is called a contraction condition. On a complete metric space, weak contraction type conditions are discussed in^41,42^. Here we define the weak contraction on a complex valued Banach space. If there exists $\mu \in (0,1)$ and $\nu \ge 0$ such that

14 $$\begin{aligned} ||Fh_{1}-Fh_{2}||\preceq \mu ||h_{1}-h_{2}||+ \nu ||h_{1}-Fh_{1}||, \end{aligned}$$

for all $h_{1}, h_{2} \in B \subseteq A$, *F* is called a weak contraction or almost weak contraction condition.

## Main results

In this section, we propose the following new iterative process for a sequence $\{\ell _n\}$ such that:

15 $$\begin{aligned} {\left\{ \begin{array}{ll} \ell _1 = \ell \in B,\\ \ell _{n+1} = F((1-a_n) u_{n}+a_n Fu_{n}), \\ u_n = Fv_n, \\ v_n = F((1-b_n) w_{n}+b_nFw_{n}), \\ w_{n} = F((1-c_n) \ell _{n}+c_n F\ell _{n}), \forall n\in {\mathbb {N}}, \end{array}\right. } \end{aligned}$$

where $\{a_n\}, \{b_n\}$ and $\{c_n\}$ are sequences in [0, 1]. This iterative process given by (15) can be called the M-Fast iterative process. In this main result, we first discuss the rate of convergence of a new iterative process named M-Fast for contraction and weak contraction conditions on complex valued metric spaces. Then we prove analytically and with numerical examples that our new four-step iterative method converges faster than other three-step iterative methods (named S-iterative, PMH, PKH, PIH, and Picard-S hybrid) and four-step iterative methods (named HR-iterative and $HR^{*}$-iterative). Furthermore, the stability of our new iterative method and the data dependence found for contraction conditions by employing our new iterative method are also discussed.

### Convergence analysis

#### Strong convergence results for our new iterative process

We initiate this section with the subsequent convergence result of the M-Fast iterative method for contraction conditions on a complex valued Banach space.

##### Theorem 3.1

Let *B* be a nonempty closed convex subset of a complex-valued Banach space (*A*, ||.||). Suppose *F* is a self-mapping on *B* satisfying the condition (13) and possessing a fixed point. Consider the iterative sequence $\{\ell _n\}$ generated by (15), where the sequences $\{a_n\}, \{b_n\}, \{c_n\}$ are real and lie within the closed interval [0, 1] such that $\sum _{n=1}^{\infty } a_n = \infty$. Then, the sequence $\{\ell _n \}$ converges strongly to a unique fixed point $\varkappa$ of the mapping *F*.

##### Proof

Let $\varkappa$ be a unique fixed point of the mapping *F*. Using (13) and (15), we have

16 $$\begin{aligned} ||w_{n}-\varkappa ||&= ||F((1-c_n) \ell _{n}+c_n F\ell _{n})-\varkappa ||\nonumber \\&\preceq \mu (1-c_n)||\ell _{n}-\varkappa ||+ \mu c_n|| F\ell _{n}-\varkappa ||\nonumber \\&\preceq \mu (1-c_n)||\ell _{n}-\varkappa ||+ \mu ^2 c_n|| \ell _{n}-\varkappa ||\nonumber \\&= \mu (1-c_n+ \mu c_n)|| \ell _{n}-\varkappa ||.\end{aligned}$$

17 $$\begin{aligned} ||v_{n}-\varkappa ||&= ||F((1-b_n) w_{n}+b_n Fw_{n})-\varkappa ||\nonumber \\&\preceq \mu (1-b_n)|| w_{n}-\varkappa ||+ \mu b_n|| F w_{n}-\varkappa ||\nonumber \\&\preceq \mu (1-b_n)|| w_{n}-\varkappa ||+ \mu ^2 b_n|| w_{n}-\varkappa ||\nonumber \\&= \mu (1-b_n+ \mu b_n)|| w_{n}-\varkappa ||.\end{aligned}$$

18 $$\begin{aligned} ||u_{n}-\varkappa ||&= ||F(v_n)-\varkappa || \preceq \mu ||v_n-\varkappa ||. \end{aligned}$$

Using (16), (17) and (18), we have

19 $$\begin{aligned} ||\ell _{n+1}-\varkappa ||&= ||F((1-a_n) u_{n}+a_n Fu_{n})-\varkappa ||\nonumber \\&\preceq \mu (1-a_n)||u_{n}-\varkappa ||+ \mu a_n|| Fu_{n}-\varkappa ||\nonumber \\&\preceq \mu (1-a_n)||u_{n}-\varkappa ||+ \mu ^2 a_n||u_{n}-\varkappa ||\nonumber \\&\preceq \mu (1-a_n+ \mu a_n)|| u_{n}-\varkappa ||\nonumber \\&\preceq \mu ^2 (1-a_n+ \mu a_n)|| v_{n}-\varkappa ||\nonumber \\&\preceq \mu ^4 (1-a_n+ \mu a_n) (1-b_n+ \mu b_n) (1-c_n+ \mu c_n)|| \ell _{n}-\varkappa || . \end{aligned}$$

Since $$0< \mu < 1$$ and $$b_n$$ and $$c_n$$$$\in [0,1]$$$$\forall$$$$n\ge 1,$$$$(1-b_n(1-\mu ))$$$$(1-c_n(1-\mu ))$$$$< 1$$. Thus the above equation (19) reduces to

20 $$\begin{aligned} ||\ell _{n+1}-\varkappa || \preceq \mu ^4 (1-a_n+ \mu a_n)|| \ell _{n}-\varkappa ||, \end{aligned}$$

where $$(1-a_n(1-\mu ))$$ is in (0, 1). As $$\mu \in (0, 1)$$ and $$a_n$$$$\in [0,1]$$ for all $$n\ge 1$$, we obtain

$$\begin{aligned} {\left\{ \begin{array}{ll} ||\ell _{n+1}-\varkappa || \preceq \mu ^4 (1-a_n+ \mu a_n) || \ell _{n}-\varkappa ||\\ ||\ell _{n}-\varkappa || \preceq \mu ^4 (1-a_{n-1}+ \mu a_{n-1}) ||\ell _{n-1}-\varkappa ||\\ .\\ .\\ .\\ ||\ell _{2}-\varkappa ||\preceq \mu ^4 (1-a_{1}+ \mu a_{1}) || \ell _{1}-\varkappa ||. \end{array}\right. } \end{aligned}$$

Therefore we have

21 $$\begin{aligned} ||\ell _{n+1}-\varkappa || \preceq ||\ell _{1}-\varkappa ||\mu ^{4(n+1)} \displaystyle \prod _{k=1}^{n}(1- a_k(1-\mu )). \end{aligned}$$

Using the classical result, $1-x \le e^{-x}$ for all $x \in [0, 1]$ in the above inequality, we get

$$||\ell _{n+1}-\varkappa || \preceq \dfrac{||\ell _{1}-\varkappa || \mu ^{4(n+1)}}{e^{(1-\mu ) \sum _{k=1}^{n} (a_k)}}$$ and $$\displaystyle \lim _{n\rightarrow \infty } |||\ell _{n+1}-\varkappa ||| \le \dfrac{||\ell _{1}-\varkappa || \mu ^{4(n+1)}}{e^{(1-\mu ) \sum _{k=1}^{n} (a_k)}} \rightarrow 0$$ as $$n\rightarrow \infty$$.

$$\implies \ell _n \rightarrow \varkappa$$ as $$n\rightarrow \infty .$$

$$\square$$

Using the aforementioned theorem technique, we provide the following result for the weak contraction condition.

##### Theorem 3.2

Let (*A*, ||.||) be a complex-valued Banach space. Given a nonempty closed convex subset $B \subseteq A$, consider a self-mapping $F: B \rightarrow B$ that satisfies condition (14). Let ${\ell _n}$ be an iterative sequence generated by (15), with real sequences $\{a_n\}, \{b_n\}, \{c_n\}$ in [0, 1] such that $\sum _{n=1}^{\infty } a_n = \infty$. Then, the sequence $\{\ell _n\}$ converges strongly to a unique fixed point $\varkappa$ of the mapping *F*.

#### Speed of convergence of our iterative process with other two- and three-step iterative processes

The following theorem shows that our new iterative process converges faster than the S-iterative, PMH, PKH , PIH, and Picard-S hybrid iterative processes.

##### Theorem 3.3

Let (*A*, ||.||) be a complex-valued normed space with *B* being a nonempty closed convex subset. Consider a self-mapping $F: B \rightarrow B$ satisfying condition (13). Assume that each iterative process defined by equations (5), (6), (7), (8), (9), and (15) converges to the same fixed point $\varkappa$ of *F*. Here, the sequences $\{a_n\}$, $\{b_n\}$, and $\{c_n\}$ are real with $0<\rho \le \gamma , a_n, b_n, c_n <1$ for all $n \in {\mathbb {N}}$. Then, the M-Fast iterative process (15) exhibits a faster convergence rate compared to all other iterative processes mentioned.

##### Proof

Let $F(\varkappa ) = \varkappa$. Use S-iterative process (5) in the contraction condition (13), by usual technique we have,

$$\begin{aligned} ||\kappa _{n}-\varkappa ||&\preceq (1-b_n)||p_{n}-\varkappa ||+b_n \mu ||p_{n}-\varkappa ||. \end{aligned}$$

Thus

$$\begin{aligned} ||p_{n+1}-\varkappa ||&= ||(1-a_n)Fp_{n}+a_n F\kappa _{n}-\varkappa || \\&\preceq (1-a_n)||Fp_{n}-\varkappa ||+a_n||F\kappa _{n}-\varkappa ||\\&\preceq (1-a_n)\mu ||p_{n}-\varkappa ||+a_n\mu ||\kappa _{n}-\varkappa ||\\&= (1-a_n)\mu ||p_{n}-\varkappa ||+a_n\mu ( (1-b_n)||p_{n}-\varkappa ||+b_n \mu ||p_{n}-\varkappa ||)\\&= \mu (1-(1-\mu )a_nb_n)||p_{n}-\varkappa ||\\&\preceq \mu (1-(1-\mu )\rho ^2)||p_{n}-\varkappa ||. \end{aligned}$$

$$||p_{n+1}-\varkappa ||\preceq \mu (1-(1-\mu )\rho ^2) ||p_{n}-\varkappa || ...\preceq \mu ^{n+1}(1-(1-\mu )\rho ^2)^{n+1}||p_{1}-\varkappa ||$$. We let $$A_n = (\mu (1-(1-\mu )\rho ^2))^{n+1}||p_{1}-\varkappa ||.$$

From Picard Mann hybrid process (6) and contraction condition (13), we have

$$\begin{aligned} ||u_{n+1}-\varkappa ||&= ||F\kappa _{n}-\varkappa || \\&\preceq \mu ||\kappa _{n}-\varkappa ||\\&= \mu ||(1-a_n)u_{n}+a_n Fu_{n}-\varkappa ||\\&\preceq \mu (1-a_n)||u_{n}-\varkappa ||+\mu a_n||Fu_{n}-\varkappa ||\\&\preceq \mu (1-a_n)||u_{n}-\varkappa ||+\mu ^2a_n||u_{n}-\varkappa ||\\&= \mu (1 - (1-\mu )a_n)||u_{n}-\varkappa ||\\&\preceq \mu (1 - (1-\mu )\rho ^2)||u_{n}-\varkappa ||. \end{aligned}$$

$$\implies$$$$||u_{n+1}-\varkappa || \preceq (\mu (1 - (1-\mu )\rho ^2))^{n+1}||u_{1}-\varkappa ||$$. Let $$B_n=(\mu (1 - (1-\mu )\rho ^2))^{n+1}||u_{1}-\varkappa ||$$.

From Picard Krasnoselskii hybrid iterative (7) and (13), we have

$$\begin{aligned} ||v_{n+1}-\varkappa ||&= ||F\kappa _{n}-\varkappa ||\\&\preceq \mu ||\kappa _{n}-\varkappa ||\\ &= \mu ||(1-\gamma )v_{n}+\gamma Fv_{n}-\varkappa || \\&\preceq \mu (1-\gamma )||v_{n}-\varkappa ||+ \mu \gamma ||Fv_{n}-\varkappa ||\\&\preceq \mu (1-\gamma )||v_{n}-\varkappa ||+ \mu ^2\gamma ||v_{n}-\varkappa ||\\&= \mu (1-\gamma + \mu \gamma ) ||v_{n}-\varkappa ||\\&\preceq \mu (1-(1- \mu )\rho ^2) ||v_{n}-\varkappa ||. \end{aligned}$$

Since $$||v_{n+1}-\varkappa || \preceq \mu (1-(1- \mu )\rho ^2) ||v_{n}-\varkappa || \preceq ....\preceq \mu ^{n+1} (1-(1- \mu )\rho ^2)^{n+1} ||v_{1}-\varkappa ||$$.

Let $$C_n = (\mu (1-(1- \mu )\rho ^2))^{n+1} ||v_{1}-\varkappa ||.$$

From Picard Ishikawa hybrid (8) and (13),

$$\begin{aligned} ||v_{n}-\varkappa ||&\preceq (1-a_n) ||\omega _{n}-\varkappa ||+\mu a_n||u_{n}-\varkappa ||.\\ ||u_{n}-\varkappa ||&\preceq (1-b_n) ||\omega _{n}-\varkappa ||+\mu b_n||\omega _{n}-\varkappa ||. \end{aligned}$$

Thus

$$\begin{aligned} ||\omega _{n+1}-\varkappa ||&= ||Fv_{n}-\varkappa || \\&\preceq \mu ||v_{n}-\varkappa ||\\&\preceq \mu (1-a_n) ||\omega _{n}-\varkappa ||+\mu ^2 a_n((1-b_n) ||\omega _{n}-\varkappa ||+\mu b_n)||\omega _{n}-\varkappa ||\\&= \mu ( (1-(1- \mu )a_n||\omega _{n}-\varkappa ||\\&\preceq \mu (1-(1- \mu )\rho ^2)||\omega _{n}-\varkappa ||. \end{aligned}$$

We have $||\omega _{n+1}-\varkappa || \preceq \mu (1-(1- \mu )\rho ^2)||\omega _{n}-\varkappa ||\preceq ....\preceq \mu ^{n+1} (1-(1- \mu )\rho ^2)^{n+1}||\omega _{1}-\varkappa ||$. Let $D_n = (\mu (1-(1- \mu )\rho ^2))^{n+1}||\omega _{1}-\varkappa ||$.

From Picard-S hybrid iterative process (9) and the condition (13),

$$\begin{aligned} ||\kappa _{n}-\varkappa ||&\preceq (1-b_n)||x_{n}-\varkappa ||+\mu b_n|| x_{n}-\varkappa ||.\\ ||z_{n}-\varkappa ||&\preceq \mu (1-a_n)||x_{n}-\varkappa ||+\mu a_n||\kappa _{n}-\varkappa ||. \end{aligned}$$

Therefore,

$$\begin{aligned} ||x_{n+1}-\varkappa ||&= ||Fz_{n}-\varkappa ||\\&\preceq \mu ||z_{n}-\varkappa ||\\&\preceq \mu (\mu (1-a_n)||x_{n}-\varkappa ||+\mu a_n||\kappa _{n}-\varkappa ||)\\&\preceq \mu (\mu (1-a_n)||x_{n}-\varkappa ||+\mu a_n((1-b_n)||x_{n}-\varkappa ||+\mu b_n|| x_{n}-\varkappa ||))\\&= \mu ^2(1- (1-\mu )a_n b_n))||x_{n}-\varkappa ||\\&\preceq \mu ^2(1- (1-\mu )\rho ^2)||x_{n}-\varkappa ||. \end{aligned}$$

$$||x_{n+1}-\varkappa ||\preceq \mu ^2(1- (1-\mu )\rho ^2)||x_{n}-\varkappa ||\preceq  ...\preceq \mu {^{2(n+1)}} (1- (1-\mu )\rho ^2))^{n+1}||x_{1}-\varkappa ||$$. So Let $$E_n = \mu ^{2(n+1)}(1- (1-\mu )\rho ^2)^{n+1}||x_{1}-\varkappa ||$$.

For M-Fast iterative process (15) and the contraction condition (13), it follows from the equation (20)

$$\begin{aligned} ||\ell _{n+1}-\varkappa ||&\preceq \mu ^4 (1-a_n+ \mu a_n)|| \ell _{n}-\varkappa ||\\&\preceq \mu ^4 (1-(1-\mu )\rho ^2) || \ell _{n}-\varkappa || \end{aligned}$$

$$||\ell _{n+1}-\varkappa ||\preceq \mu ^4(1- (1-\mu )\rho ^2) ||\ell _{n}-\varkappa ||\preceq ...\preceq \mu ^{4(n+1)}(1- (1-\mu )\rho ^2)^{n+1}||\ell _{1}-\varkappa ||$$.

Let $$F_n = (\mu ^4(1- (1-\mu )\rho ^2))^{n+1}||\ell _{1}-\varkappa ||$$.

Now we show that rate of convergence. Since $(\mu (1- (1-\mu )\rho ^2)) < 1$ and $\mu \in (0, 1)$,

we observe the following,

$$\frac{F_n}{A_n} = \dfrac{(\mu ^4(1- (1-\mu )\rho ^2))^{n+1}||\ell _{1}-\varkappa ||}{(\mu (1-(1-\mu )\rho ^2))^{n+1}||p_{1}-\varkappa ||} = \mu ^{3(n+1)} \dfrac{||\ell _{1}-\varkappa ||}{||p_{1}-\varkappa ||}.$$

Letting $n\rightarrow \infty$, we have $\lim _{n\rightarrow \infty }\frac{F_n}{A_n} = 0$. Thus M-Fast iterative process (15) converges to $\varkappa$ faster than S-iterative process (5).

$$\frac{F_n}{B_n} = \dfrac{(\mu ^4(1- (1-\mu )\rho ^2))^{n+1}||\ell _{1}-\varkappa ||}{(\mu (1 - (1-\mu )\rho ^2))^{n+1}||u_{1}-\varkappa ||} = \mu ^{3(n+1)} \dfrac{||\ell _{1}-\varkappa ||}{||u_{1}-\varkappa ||}.$$

As $n\rightarrow \infty$, we have $\lim _{n\rightarrow \infty }\frac{F_n}{B_n} = 0$. Thus M-Fast iterative process (15) converges to $\varkappa$ faster than Picard Mann hybrid process (6).

$$\frac{F_n}{C_n} = \dfrac{(\mu ^4(1- (1-\mu )\rho ^2))^{n+1}||\ell _{1}-\varkappa ||}{(\mu (1 - (1-\mu )\rho ^2))^{n+1}||v_{1}-\varkappa ||}= \mu ^{3(n+1)} \dfrac{||\ell _{1}-\varkappa ||}{||v_{1}-\varkappa ||}.$$

Letting $n\rightarrow \infty$, we have $\lim _{n\rightarrow \infty }\frac{F_n}{C_n} = 0$. Thus M-Fast iterative process (15) converges to $\varkappa$ faster Picard Krasnoselskii hybrid iterative (7).

$$\frac{F_n}{D_n} = \dfrac{(\mu ^4(1- (1-\mu )\rho ^2))^{n+1}||\ell _{1}-\varkappa ||}{(\mu (1-(1- \mu )\rho ^2))^{n+1}||w_{1}-\varkappa ||} = \mu ^{3(n+1)} \dfrac{||\ell _{1}-\varkappa ||}{||w_{1}-\varkappa ||.}$$

As $n\rightarrow \infty$, we have $\lim _{n\rightarrow \infty }\frac{F_n}{D_n} = 0$. Thus M-Fast iterative process (15) converges to $\varkappa$ faster Picard Ishikawa hybrid iterative (8).

$$\frac{F_n}{E_n} = \dfrac{(\mu ^4(1- (1-\mu )\rho ^2))^{n+1}||\ell _{1}-\varkappa ||}{(\mu ^2 (1-(1- \mu )\rho ^2))^{n+1} ||x_{1}-\varkappa ||} = \mu ^{3(n+1)} \dfrac{||\ell _{1}-\varkappa ||}{||x_{1}-\varkappa ||}$$

Letting $$n\rightarrow \infty$$, we have $$\lim _{n\rightarrow \infty }\frac{F_n}{E_n} = 0$$. Thus M-Fast iterative process (15) converges to $$\varkappa$$ faster Picard-S hybrid (PSH) iterative process (9). Therefore $$\{\ell _n\}$$ converges faster than $$\{p_n\}$$, $$\{u_n\}$$,  $$\{v_n\}$$$$\{w_n\}$$ and $$\{x_n\}$$. That is the M-Fast iterative process (15) exhibits a faster convergence rate compared to all other iterative processes mentioned. $$\square$$

Based on the technique mentioned earlier, we prove the following theorem under the condition of weak contraction.

##### Theorem 3.4

Let (*A*, ||.||) a complex valued normed space and *B* be a nonempty closed convex subset of (*A*, ||.||). Let *F* be a mapping from *B* to *B* satisfying condition (14). Assume that each iterative process in (5), (6), (7), (8), (9) and (15) converges to the same fixed point $$\varkappa$$ of *F* where $$\{a_n\}, \{b_n\}$$ and $$\{c_n\}$$ are the real sequences in $$0<\rho \le \gamma , a_n, b_n, c_n <1$$$$\forall n \in {\mathbb {N}}$$. Then M-Fast iterative process (15) exhibits a faster convergence rate compared to all other iterative processes mentioned.

Here we provide the following example to ensure that the analytical proof in the above theorem is valid.

##### Example 1

Let $A = {\mathbb {R}}$ and $B = [1, 10]$. Let $F: [1, 10] \rightarrow [1, 10]$ be a self operator which is defined by

$$F(x) = \root 4 \of {7x+2}$$, $$\forall x \in B$$.

**Fig. 1:**
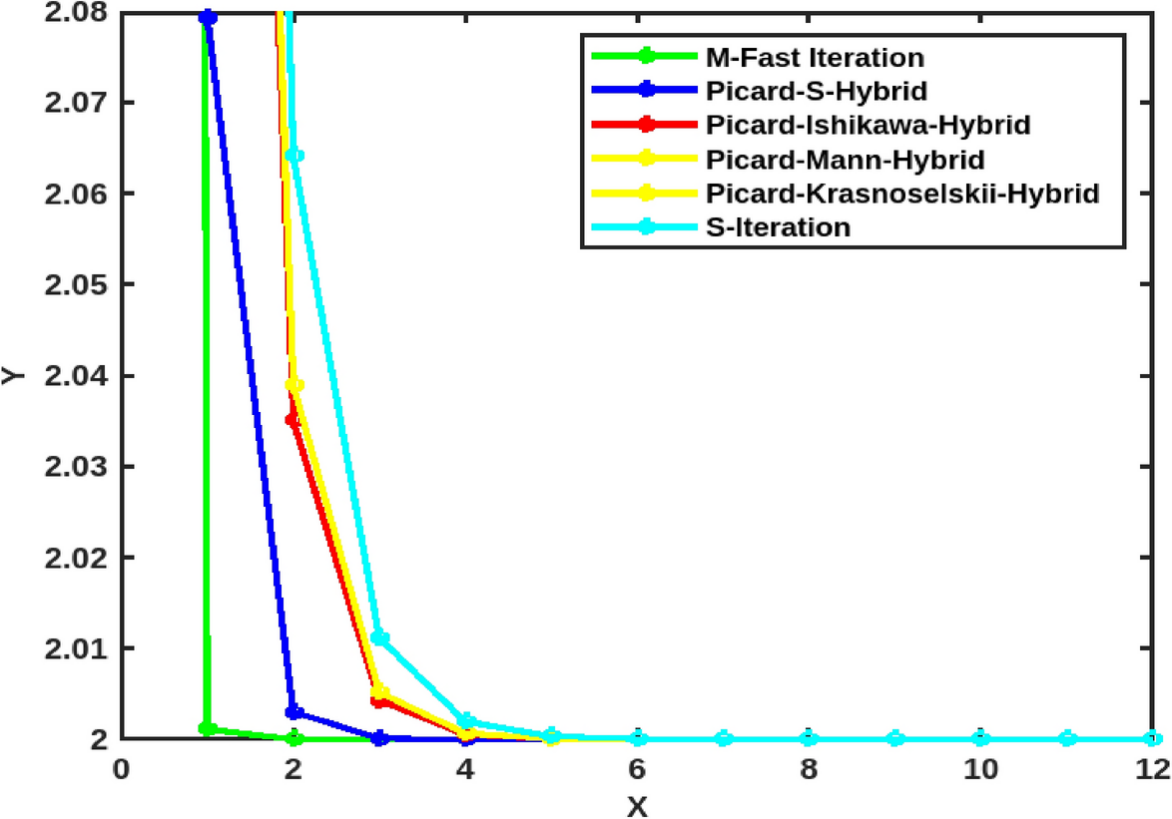
Comparison of the speed of convergence of M-Fast iteration process with the other two- and three- step iterative processes.

Taking $\gamma = a_n = b_n = c_n = \frac{1}{2}$ for $n\in {\mathbb {N}}$, with initial values $x_0 = 5$, it satisfies condition (13) for $\mu = \frac{1}{\root 4 \of {2}}$ and also condition (14) when $\nu = 0.$ Therefore, it has a unique fixed point $\varkappa =2$. From Table 1 and Fig. 1, we can see that the M-Fast iterative process (15) exhibits a faster convergence rate compared to all other iterative processes mentioned.

**Table 1:** Comparison of the speed of convergence of the M-Fast iterative process with the other two and three step iterative processes.

Step	M-Fast Iterative	Picard-S-Hybrid	Picard-Ishikawa-Hybrid	Picard-Mann-Hybrid	Picard-Krasnoselskii Hybrid	S-Iteration
0	5.0000000000000000	5.0000000000000000	5.0000000000000000	5.0000000000000000	5.0000000000000000	5.0000000000000000
1	2.0011261867836319	2.0793059134079650	2.2912488865942713	2.3030358186374400	2.3030358186374400	2.3846807665985503
2	2.0000005834225019	2.0030110496598703	2.0350206591613547	2.0389097369451790	2.0389097369451790	2.0641149195728792
3	2.0000000003022937	2.0001158794067488	2.0043246344827916	2.0051608040420277	2.0051608040420277	2.0111783140030237
4	2.0000000000001563	2.0000044619078006	2.0005358084723226	2.0006874802484398	2.0006874802484398	2.0019643560533171
5	2.0000000000000000	2.0000001718080949	2.0000664121678637	2.0000916335015981	2.0000916335015981	2.0003456740638228
6	2.0000000000000000	2.0000000066155659	2.0000082320460413	2.0000122146724939	2.0000122146724939	2.0000608442374688
7	2.0000000000000000	2.0000000002547358	2.0000010204004890	2.0000016282228121	2.0000016282228121	2.0000107100323050
8	2.0000000000000000	2.0000000000098086	2.0000001264834970	2.0000002170433273	2.0000002170433273	2.0000018852345725
9	2.0000000000000000	2.0000000000003775	2.0000000156782329	2.0000000289320443	2.0000000289320443	2.0000003318490576
10	2.0000000000000000	2.0000000000000147	2.0000000019433917	2.0000000038566639	2.0000000038566639	2.0000000584138569
11	2.0000000000000000	2.0000000000000004	2.0000000002408926	2.0000000005140963	2.0000000005140963	2.0000000102823217
12	2.0000000000000000	2.0000000000000000	2.0000000000298597	2.0000000000685292	2.0000000000685292	2.0000000018099495
13	2.0000000000000000	2.0000000000000000	2.0000000000037010	2.0000000000091349	2.0000000000091349	2.0000000003185967
14	2.0000000000000000	2.0000000000000000	2.0000000000004587	2.0000000000012177	2.0000000000012177	2.0000000000560814
15	2.0000000000000000	2.0000000000000000	2.0000000000000568	2.0000000000001621	2.0000000000001621	2.0000000000098717
16	2.0000000000000000	2.0000000000000000	2.0000000000000071	2.0000000000000213	2.0000000000000213	2.0000000000017373
17	2.0000000000000000	2.0000000000000000	2.0000000000000009	2.0000000000000027	2.0000000000000027	2.0000000000003055
18	2.0000000000000000	2.0000000000000000	2.0000000000000000	2.0000000000000004	2.0000000000000004	2.0000000000000533
19	2.0000000000000000	2.0000000000000000	2.0000000000000000	2.0000000000000000	2.0000000000000000	2.0000000000000093
20	2.0000000000000000	2.0000000000000000	2.0000000000000000	2.0000000000000000	2.0000000000000000	2.0000000000000013

#### Speed of convergence of M-Fast iterative process with other four- step iterative processes

Now, we prove that the new iterative process converges faster than the other four-step iterative processes, namely *HR* and $HR^*$, on a complex valued normed space.

##### Theorem 3.5

Let *F* be a self-mapping on a nonempty closed convex subset *B* of a complex valued normed space (*A*, ||.||) that satisfies the condition (13). Assume that each iterative process in (11), (12), and (15) converges to the same fixed point $$\varkappa$$ of *F*, where $$\{a_n\}, \{b_n\}$$, and $$\{c_n\}$$ are the real sequences with $$0<\rho \le a_n, b_n, c_n <1$$$$\forall$$$$n \in {\mathbb {N}}$$. Then the M-Fast iterative process (15) exhibits a faster convergence rate compared to all other iterative processes mentioned.

##### Proof

For M-Fast iterative process (15) and the contraction condition (13), it follows from the equation (21) together with $0<\rho \le a_n <1$, $n \in {\mathbb {N}}$,

$$\begin{aligned} ||\ell _{n+1}-\varkappa ||&\preceq ||\ell _{1}-\varkappa ||\mu ^{4(n+1)} \displaystyle \prod _{k=1}^{n}(1- \rho (1-\mu ))\\&= ||\ell _{1}-\varkappa ||\mu ^{4(n+1)} (1- \rho (1-\mu ))^{4(n+1)} \end{aligned}$$

Let $$F_n = (\mu ^4(1- (1-\mu )\rho ))^{n+1}||\ell _{1}-\varkappa ||$$.

For *HR* iterative process (11) and the contraction condition (13),

$$\begin{aligned} ||\kappa _{n}-\varkappa ||&= ||(1-a_n)z_{n}+a_n Fz_{n}-\varkappa ||\\&\preceq (1-a_n)||z_{n}-\varkappa ||+a_n ||F(z_{n})-\varkappa ||\\&\preceq (1-a_n)||z_{n}-\varkappa ||+ \mu a_n ||z_{n}-\varkappa || \\&\preceq (1-a_n+\mu a_n)||z_{n}-\varkappa ||. \end{aligned}$$

Similarly, we have $$||y_{n}-\varkappa || \preceq \mu (1-c_n+\mu c_n)||x_{n}-\varkappa ||,$$$$||x_{n}-\varkappa || \preceq \mu (1-b_n+\mu b_n)||\kappa _{n}-\varkappa ||$$. Thus

22 $$\begin{aligned} ||z_{n+1}-\varkappa ||&= ||Fy_{n}-\varkappa ||\nonumber \\ &\preceq \mu ||y_{n}-\varkappa ||\nonumber \\&\preceq \mu ^2 (1-c_n+\mu c_n)||x_{n}-\varkappa ||\nonumber \\ &\preceq \mu ^3 (1-c_n+\mu c_n)(1-b_n+\mu b_n)||\kappa _{n}-\varkappa ||\nonumber \\&\preceq \mu ^3 (1-a_n+\mu a_n) (1-b_n+\mu b_n) (1-c_n+\mu c_n)||z_{n}-\varkappa ||. \end{aligned}$$

Since $$\mu$$ in (0, 1) and $$b_n$$, $$c_n$$ are in [0, 1] $$\forall n \in {\mathbb {N}}$$, we get $$(1-b_n(1-\mu ))$$$$(1-c_n(1-\mu ))$$$$< 1$$. Thus above inequality (22) reduces to

23 $$\begin{aligned} ||z_{n+1}-\varkappa || \preceq \mu ^3 (1-a_n+\mu a_n) ||z_{n}-\varkappa ||, \end{aligned}$$

where $$(1-a_n(1-\mu ))$$ is in (0, 1). Since $$\mu \in (0, 1)$$ and $$a_n$$$$\in [0,1]$$ for all $$n \in {\mathbb {N}}$$, we obtain

24 $$\begin{aligned} ||z_{n+1}-\varkappa || \preceq ||z_{1}-\varkappa ||\mu ^{3(n+1)} \displaystyle \prod _{k=1}^{n}(1- a_k(1-\mu )). \end{aligned}$$

It follows from equation (24) together with $$0<\rho \le a_n <1$$$$\forall n \in {\mathbb {N}}$$,

$$\begin{aligned} ||z_{n+1}-\varkappa ||&\preceq ||z_{1}-\varkappa ||\mu ^{3(n+1)} \displaystyle \prod _{k=1}^{n}(1- \rho (1-\mu ))\\&= ||z_{1}-\varkappa ||\mu ^{3(n+1)} (1- \rho (1-\mu ))^{3(n+1)} \end{aligned}$$

Let $$G_n = (\mu ^3(1- (1-\mu )\rho ))^{n+1}||z_{1}-\varkappa ||$$.

For $HR^*$ iterative process (12) and the contraction condition (13), we got $||\kappa _{n}-\varkappa ||\preceq (1-a_n+\mu a_n)||o_{n}-\varkappa ||$ and $|x_{n}-\varkappa || \preceq \mu (1-b_n+\mu b_n)||\kappa _{n}-\varkappa ||.$

$$\begin{aligned} ||z_{n}-\varkappa ||&=||F(F(x_n))-\varkappa ||\\&\preceq \mu ^2 ||x_n-\varkappa ||. \end{aligned}$$

Thus

$$\begin{aligned} ||o_{n}-\varkappa ||&= ||(1-c_n)z_{n}+c_n Fz_{n}-\varkappa ||\\&\preceq (1-c_n)||z_{n}-\varkappa ||+c_n ||F(z_{n})-\varkappa ||\\&\preceq (1-c_n)||z_{n}-\varkappa ||+ \mu c_n ||z_{n}-\varkappa || \\&\preceq (1-c_n+\mu c_n)||z_{n}-\varkappa || \\&\preceq \mu ^3 (1-c_n+\mu c_n)(1-b_n+\mu b_n)||\kappa _{n}-\varkappa || \\&\preceq \mu ^3 (1-c_n+\mu c_n)(1-b_n+\mu b_n)(1-a_n+\mu a_n)||o_{n}-\varkappa ||\\&\preceq \mu ^3 (1-a_n+\mu a_n)||o_{n}-\varkappa ||, \end{aligned}$$

where $$(1-a_n(1-\mu ))$$ is in (0, 1). As $$\mu \in (0, 1)$$ and $$a_n$$$$\in [0,1]$$$$\forall n \in {\mathbb {N}}$$, we find

25 $$\begin{aligned} ||o_{n+1}-\varkappa || \preceq ||o_{1}-\varkappa ||\mu ^{3(n+1)} \displaystyle \prod _{k=1}^{n}(1- a_k(1-\mu )). \end{aligned}$$

It follows from the equation (25) together with $$0<\rho \le a_n <1$$$$\forall n \in {\mathbb {N}}$$,

$$\begin{aligned} ||o_{n+1}-\varkappa ||&\preceq ||o_{1}-\varkappa ||\mu ^{3(n+1)} \displaystyle \prod _{k=1}^{n}(1- \rho (1-\mu ))\\&= ||o_{1}-\varkappa ||\mu ^{3(n+1)} (1- \rho (1-\mu ))^{3(n+1)}. \end{aligned}$$

Let $H_n = (\mu ^3(1- (1-\mu )\rho ))^{n+1}||o_{1}-\varkappa ||$. While checking the rate of convergence, we observe the following,

$$\frac{F_n}{G_n} = \dfrac{(\mu ^4(1- (1-\mu )\rho ))^{n+1}||\ell _{1}-\varkappa ||}{(\mu ^3 (1-(1-\mu )\rho ))^{n+1}||z_{1}-\varkappa ||} = \mu ^{(n+1)} \dfrac{||\ell _{1}-\varkappa ||}{||z_{1}-\varkappa ||},$$

since $(\mu (1- (1-\mu )\rho )) < 1$ and $\mu \in (0, 1)$ Letting $n\rightarrow \infty$, we have $\lim _{n\rightarrow \infty }\frac{F_n}{G_n} = 0$. Similarly,

$$\frac{F_n}{H_n} = \dfrac{(\mu ^4(1- (1-\mu )\rho ))^{n+1}||\ell _{1}-\varkappa ||}{(\mu ^3 (1-(1-\mu )\rho ))^{n+1}||o_{1}-\varkappa ||} = \mu ^{(n+1)} \dfrac{||\ell _{1}-\varkappa ||}{||o_{1}-\varkappa ||}.$$

Letting $n\rightarrow \infty$, we have $\lim _{n\rightarrow \infty }\frac{F_n}{H_n} = 0$. Thus M-Fast iterative process (15) converges to $\varkappa$ faster than *HR*-iterative process (11) and $HR^*$-iterative process (12). $\square$

The following theorem, which pertains to the condition of the weak contraction, has been proved by us, using the technique that has been discussed earlier.

##### Theorem 3.6

Let (*A*, ||.||) be a complex-valued normed space with *B* as a nonempty closed convex subset. Consider a self-mapping $$F: B \rightarrow B$$ satisfying condition (14). Suppose that every iterative process defined by equations (11), (12), and (15) converges to the same fixed point $$\varkappa$$ of *F*, where $$\{a_n\}$$, $$\{b_n\}$$, and $$\{c_n\}$$ are real sequences such that $$0< \rho \le a_n, b_n, c_n < 1$$$$\forall$$$$n \in {\mathbb {N}}$$. Then, the M-Fast iterative process (15) achieves a faster rate of convergence compared to all other iterations.

In order to show the validity of the analytical proof presented in the above Theorem, we provide a numerical illustration as follows.

##### Example 2

**Fig. 2:**
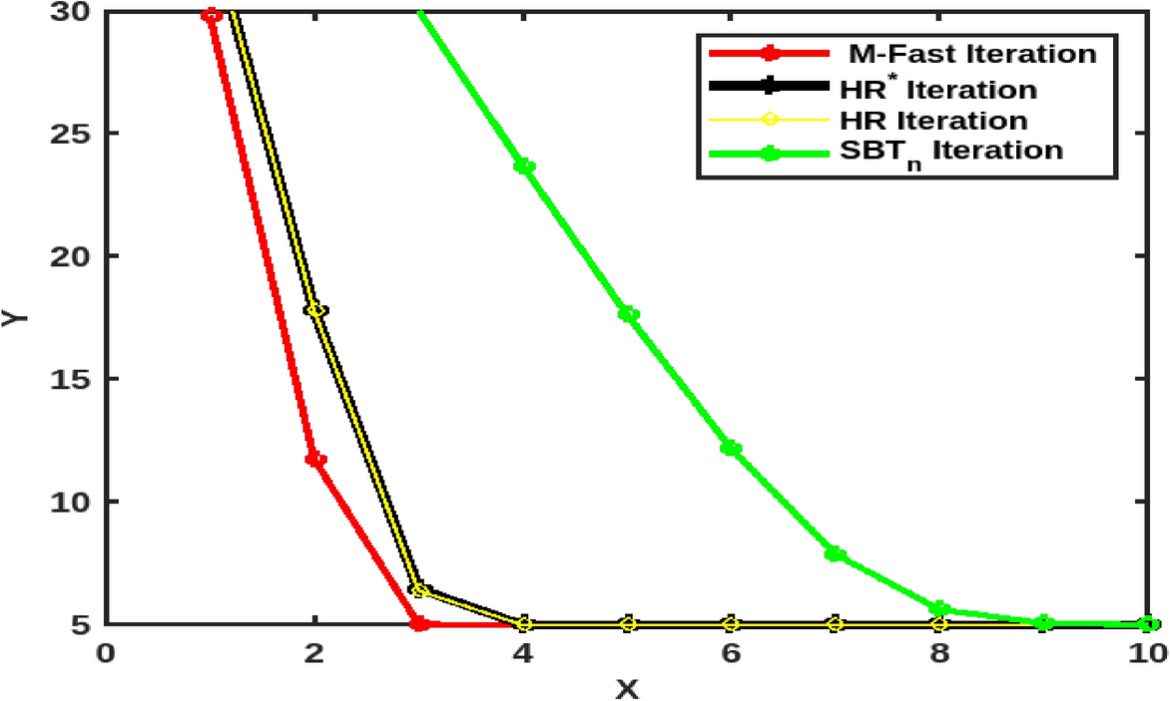
Comparison of the speed of convergence of M-Fast iterative process with the other 4-step iterative processes.

Let $A = {\mathbb {R}}$ and $B = [1, 50]$. Let $F: [1, 50] \rightarrow [1, 50]$ be a self operator which is defined by $F(x) = \sqrt{x^2-8x+40}$, $\forall x \in B$. Taking $a_n = b_n = c_n = \frac{1}{2}$ for $n\in {\mathbb {N}}$, with initial values $x_0 = 50$, it satisfies condition (13) and also (14) condition when $\nu = 0.$ Therefore, it has a unique fixed point $\varkappa =5$. From Table 2 and Fig. 2, we can see that the M-Fast iterative process (15) converges faster than all the other iterations.

**Table 2:** Comparison of the speed of convergence of the M-Fast iterative process with the other 4-step iterative processes.

Step	M-Fast iteration	$HR^*$ iteration	HR iteration	$SBT_n$ iteration
0	50.0000000000000000	50.0000000000000000	50.0000000000000000	50.0000000000000000
1	29.7983219777272730	33.3923263795198508	33.3904475851243419	43.2444875224078089
2	11.7418515288816714	17.8112141702975144	17.7997315173083202	36.5781382065469671
3	5.0322485948855791	6.4758736098510514	6.3988545945311150	30.0377438189666250
4	5.0000112969950772	5.0043238443811520	5.0038977070800321	23.6868656427377005
5	5.0000000039042600	5.0000074826778516	5.0000067439454190	17.6468208208834980
6	5.0000000000013491	5.0000000129301005	5.0000000116535634	12.1787126296530488
7	5.0000000000000000	5.0000000000223430	5.0000000000201377	7.8808153287967073
8	5.0000000000000000	5.0000000000000382	5.0000000000000346	5.6284225798452923
9	5.0000000000000000	5.0000000000000000	5.0000000000000000	5.0751181454845957
10	5.0000000000000000	5.0000000000000000	5.0000000000000000	5.0072852038527920
11	5.0000000000000000	5.0000000000000000	5.0000000000000000	5.0006869105740304
12	5.0000000000000000	5.0000000000000000	5.0000000000000000	5.0000645882705879
13	5.0000000000000000	5.0000000000000000	5.0000000000000000	5.0000060714625523
14	5.0000000000000000	5.0000000000000000	5.0000000000000000	5.0000005707189388
15	5.0000000000000000	5.0000000000000000	5.0000000000000000	5.0000000536475930
16	5.0000000000000000	5.0000000000000000	5.0000000000000000	5.0000000050428746
17	5.0000000000000000	5.0000000000000000	5.0000000000000000	5.0000000004740306
18	5.0000000000000000	5.0000000000000000	5.0000000000000000	5.0000000000445590
19	5.0000000000000000	5.0000000000000000	5.0000000000000000	5.0000000000041886
20	5.0000000000000000	5.0000000000000000	5.0000000000000000	5.0000000000003944

Next, we provide a small result of nonexpansive mapping for the M-Fast iterative process on a complex valued banach space.

##### Theorem 3.7

Let *F* be a nonexpansive self-mapping from a nonempty closed convex subset of a complex valued Banach space (*A*, ||.||) to itself. Assume that $\{\ell _n\}_{n=0}^{\infty }$ is generated by process(15), then $\displaystyle \lim _{n\rightarrow \infty }\Vert {\ell _n-\varkappa } \Vert$ exist $\forall \varkappa \in F(F)$.

##### Proof

Suppose $$\varkappa \in F(F)$$.

$$\begin{aligned} ||w_{n}-\varkappa ||&= ||F((1-c_n) \ell _{n}+c_n F\ell _{n})-\varkappa ||\\&\preceq (1-c_n)||\ell _{n}-\varkappa ||+ c_n|| F\ell _{n}-\varkappa ||\\&\preceq (1-c_n)||\ell _{n}-\varkappa ||+ c_n|| \ell _{n}-\varkappa ||\\&\preceq || \ell _{n}-\varkappa ||.\\ ||v_{n}-\varkappa ||&= ||F((1-b_n) w_{n}+b_n Fw_{n})-\varkappa ||\\&\preceq (1-b_n)|| w_{n}-\varkappa ||+ b_n|| F w_{n}-\varkappa ||\\&\preceq (1-b_n)|| w_{n}-\varkappa ||+ b_n|| w_{n}-\varkappa ||\\&\preceq || w_{n}-\varkappa ||.\\ ||u_{n}-\varkappa ||&= ||F(v_n)-\varkappa || \preceq ||v_n-\varkappa ||. \end{aligned}$$

Thus

$$\begin{aligned} ||\ell _{n+1}-\varkappa ||&= ||F((1-a_n) u_{n}+a_n Fu_{n})-\varkappa ||\\&\preceq (1-a_n)||u_{n}-\varkappa ||+ a_n|| Fu_{n}-\varkappa ||\\&\preceq (1-a_n)||u_{n}-\varkappa ||+ a_n||u_{n}-\varkappa ||\\&\preceq ||u_{n}-\varkappa || \preceq ||v_{n}-\varkappa || \preceq || w_{n}-\varkappa || \preceq || \ell _{n}-\varkappa ||. \end{aligned}$$

Take $_n = \ell _n-\varkappa$ for all n $\in {\mathbb {N}}$, as $\Vert {S_{n+1}} \Vert \preceq \Vert {S_{n}} \Vert$, $\displaystyle \lim _{n\rightarrow \infty }\Vert {\ell _n-\varkappa } \Vert$ exist $\forall \varkappa \in F(F)$. $\square$

### Stability results for our new iterative process in complex valued banach spaces

In this part, we prove the stability of the M-Fast iteration procedures for contraction mapping on a complex-valued Banach space.

#### Theorem 3.8

Let $(A, \Vert {.} \Vert )$ be a complex valued Banach space and $F: B \subseteq A \rightarrow B$ be a mapping that satisfies the contraction condition (13). Assume that there exists $\varkappa$ in *F*(*F*) and the sequence $\{\ell _n\}$ of (15) converges to $\varkappa$ with $\displaystyle \sum _{n=1}^{\infty } a_n = \infty$ and real sequences $0< \rho \le a_n, b_n, c_n <1$ for all $n \in {\mathbb {N}}$. Then the M-Fast iterative process is *F*-stable and almost *F*-stable.

#### Proof

Suppose $\{\ell _n\}_{n=0}^{\infty }$ in *B* is a bounded sequence and put $\epsilon _n = \Vert {\ell _{n+1}-f(F, \ell _n)} \Vert$, where

$$\begin{aligned} \ell _{n+1}&= F((1-a_n) u_{n}+a_n Fu_{n}), \\ u_n&= Fv_n, \\ v_n&= F((1-b_n) w_{n}+b_nFw_{n}), \\ w_{n}&= F((1-c_n) \ell _{n}+c_n F\ell _{n}). \end{aligned}$$

Let $\displaystyle \lim _{n\rightarrow \infty }\epsilon _n = 0$. Using (13) and (15), we have

$$\begin{aligned} \Vert {\ell _{n+1}-\varkappa } \Vert&\preceq \Vert {\ell _{n+1}-f(F, \ell _n)} \Vert +\Vert {f(F, \ell _n)-\varkappa } \Vert \\ &\preceq \epsilon _n+\Vert {F((1-a_n) u_{n}+a_n Fu_{n})-\varkappa } \Vert \\&\preceq \epsilon _n+\mu (1-a_n)||u_{n}-\varkappa ||+ \mu a_n|| Fu_{n}-\varkappa ||\\&= \epsilon _n+\mu (1-a_n+\mu a_n)||u_{n}-\varkappa ||\\&= \epsilon _n+\mu (1-a_n+\mu a_n)||Fv_{n}-\varkappa ||\\&\preceq \epsilon _n+\mu ^2 (1-a_n+\mu a_n)||v_{n}-\varkappa ||\\&= \epsilon _n+\mu ^2 (1-a_n+\mu a_n)||F((1-b_n) w_{n}+b_nFw_{n})-\varkappa ||\\&\preceq \epsilon _n+\mu ^3 (1-a_n+\mu a_n)((1-b_n)|| w_{n}-\varkappa ||+b_n||Fw_{n}-\varkappa ||)\\&\preceq \epsilon _n+\mu ^3 (1-a_n+\mu a_n)((1-b_n)|| w_{n}-\varkappa ||+\mu b_n||w_{n}-\varkappa ||)\\&= \epsilon _n+\mu ^3(1-a_n+\mu a_n)(1-b_n+ \mu b_n)|| F((1-c_n) \ell _{n}+c_n F\ell _{n})-\varkappa ||\\&\preceq \epsilon _n+\mu ^3(1-a_n+\mu a_n)(1-b_n+ \mu b_n) (\mu (1-c_n)||\ell _{n}-\varkappa ||+ \mu ^2 c_n|| \ell _{n}-\varkappa ||)\\&\preceq \epsilon _n+\mu ^4(1-a_n+\mu a_n)(1-b_n+ \mu b_n) (1-c_n+ \mu c_n)|| \ell _{n}-\varkappa ||\\&\preceq \epsilon _n+\mu ^4(1-\rho +\mu \rho )^3|| \ell _{n}-\varkappa ||. \end{aligned}$$

26 $$\begin{aligned} \Vert {\ell _{n+1}-\varkappa } \Vert \preceq \epsilon _n+\mu ^4(1-\rho +\mu \rho )^3|| \ell _{n}-\varkappa ||. \end{aligned}$$

By our assumption, we have $1-(1 - \mu ) \rho <1$. From Lemma 2.3, $\displaystyle \lim _{n\rightarrow \infty }\ell _n =\varkappa$. And then conversely

$$\begin{aligned} \epsilon _n&=\Vert {\ell _{n+1}-f(F, \ell _n)} \Vert \\&\preceq \Vert {\ell _{n+1}-\varkappa } \Vert +\Vert {\varkappa -f(F, \ell _n)} \Vert \\&\preceq \Vert {\ell _{n+1}-\varkappa } \Vert +\mu (1-a_n)||u_{n}-\varkappa ||+ \mu a_n|| Fu_{n}-\varkappa ||\\&=\Vert {\ell _{n+1}-\varkappa } \Vert + \mu (1-a_n+ \mu a_n)|| u_{n}-\varkappa ||\\&=\Vert {\ell _{n+1}-\varkappa } \Vert + \mu (1-a_n+ \mu a_n)|| Fv_{n}-\varkappa ||\\&\preceq \Vert {\ell _{n+1}-\varkappa } \Vert + \mu ^2 (1-a_n+ \mu a_n)|| v_{n}-\varkappa ||\\&=\Vert {\ell _{n+1}-\varkappa } \Vert + \mu ^2 (1-a_n+ \mu a_n)|| F((1-b_n) w_{n}+b_nFw_{n})-\varkappa ||\\&\preceq \Vert {\ell _{n+1}-\varkappa } \Vert + \mu ^3 (1-a_n+ \mu a_n)(1-b_n+ \mu b_n)||w_{n}-\varkappa ||\\&=\Vert {\ell _{n+1}-\varkappa } \Vert + \mu ^3 (1-a_n+ \mu a_n)(1-b_n+ \mu b_n)||F((1-c_n) \ell _{n}+c_n F\ell _{n})-\varkappa ||\\&\preceq \Vert {\ell _{n+1}-\varkappa } \Vert +\mu ^4 (1-a_n+ \mu a_n) (1-b_n+ \mu b_n) (1-c_n+ \mu c_n)|| \ell _{n}-\varkappa ||. \end{aligned}$$

27 $$\begin{aligned} \epsilon _n \preceq \Vert {\ell _{n+1}-\varkappa } \Vert +\mu ^4 (1-a_n+ \mu a_n) (1-b_n+ \mu b_n) (1-c_n+ \mu c_n)|| \ell _{n}-\varkappa ||. \end{aligned}$$

Therefore $\displaystyle \lim _{n\rightarrow \infty }\epsilon _n = 0$, so the M-Fast iterative process is *F*-stable . We find that the process is almost *F*-stable. Suppose $\displaystyle \sum _{n=1}^{\infty }\epsilon _n < \infty$. Using (26) we have

$$\Vert {\ell _{n+1}-\varkappa } \Vert \preceq \epsilon _n+\mu ^4(1-\rho +\mu \rho )^3|| \ell _{n}-\varkappa ||.$$

By Lemma (2.1) and (2.4), we get $\ell _{n}\rightarrow \varkappa$ as n $\rightarrow \infty$.

Conversely, suppose that $\displaystyle \lim _{n\rightarrow \infty }\ell _n = \varkappa$. From (27), we have

$$\begin{aligned} \epsilon _n \preceq \Vert {\ell _{n+1}-\varkappa } \Vert +\mu ^4 (1-a_n+ \mu a_n) (1-b_n+ \mu b_n) (1-c_n+ \mu c_n)|| \ell _{n}-\varkappa ||. \end{aligned}$$

we obtain, $\epsilon _{n}\rightarrow 0$ as n $\rightarrow \infty$. Hence the proof.

$$\square$$

#### Example 3

Let $F: [0, 1] \rightarrow [0, 1]$ be a self mapping defined by $||x-y|| = i ||x-y||$ such that $F(x) = \frac{x}{2}$. It can be checked that the condition (13) is satisfied for $\mu = \frac{1}{2}$ and $\varkappa = 0$. Suppose $\ell _{n} = \frac{1}{n}$ with $a_{n} = b_{n} = c_{n} = \frac{1}{\sqrt{2}}$.

Using (26), we have

$$\begin{aligned} \Vert {\ell _{n+1}-\varkappa } \Vert&\preceq |\epsilon _n|+|\mu ^4(1-\rho +\mu \rho )^3|| \ell _{n}-\varkappa |||\\&= |\epsilon _n|+|(\frac{1}{2})^4(1-\frac{1}{\sqrt{2}}+(\frac{1}{2}) \frac{1}{\sqrt{2}})^3||\frac{1}{n}-\varkappa |||\\&= |\epsilon _n|+|(\frac{1}{2})^4(1-\frac{1}{\sqrt{2}}+(\frac{1}{2}) \frac{1}{\sqrt{2}})^{3} i|\frac{1}{n}-0|| \rightarrow \hbox { 0 as n}\ \rightarrow \infty . \end{aligned}$$

Using (27),

$$\begin{aligned} \epsilon _n&\preceq \Vert {\ell _{n+1}-\varkappa } \Vert +\mu ^4 (1-a_n+ \mu a_n) (1-b_n+ \mu b_n) (1-c_n+ \mu c_n)|| \ell _{n}-\varkappa ||\\&\le |\Vert {\frac{1}{n+1}-\varkappa } \Vert |+|(\frac{1}{2})^4 (1-a_n+ \frac{1}{2} a_n) (1-b_n+ \frac{1}{2} b_n) (1-c_n+ \frac{1}{2} c_n)|| \ell _{n}-\varkappa |||\\&\le i|\frac{1}{n+1}-0|+|(\frac{1}{2})^4 (1-\frac{1}{\sqrt{2}}+ \frac{1}{2} \frac{1}{\sqrt{2}}) (1-\frac{1}{\sqrt{2}}+ \frac{1}{2} \frac{1}{\sqrt{2}}) (1-\frac{1}{\sqrt{2}}+ \frac{1}{2} \frac{1}{\sqrt{2}}) i| \frac{1}{n}-0||\rightarrow \hbox { 0 as n}\ \rightarrow \infty . \end{aligned}$$

$\displaystyle \lim _{n\rightarrow \infty }\epsilon _n = 0$. Therefore, the M-Fast iterative process is *F*-stable and also almost *F*-stable.

In the same line, we prove the stability of the M-Fast iteration procedures for weak contraction mapping on a complex-valued Banach space.

#### Theorem 3.9

Let $$(A, \Vert {.} \Vert )$$ be a complex valued Banach space and $$F: B \subseteq A \rightarrow B$$ be a mapping that satisfies the contraction condition (14). Assume that $$\exists$$$$\varkappa$$ in *F*(*F*),  such that the sequence $$\{\ell _n\}$$ of (15) converges to $$\varkappa$$ with $$\displaystyle \sum _{n=1}^{\infty } a_n = \infty$$ and real sequences $$0< \rho \le a_n, b_n, c_n <1$$$$\forall n \in {\mathbb {N}}$$, then the M-Fast iterative process is *F*-stable and almost *F*-stable.

### Data dependence result for our new iterative process in complex valued banach spaces

In this section, we prove the data dependence result of M- Fast iterative process for contraction mapping on a complex valued banach space.

#### Theorem 3.10

Let $\overset{\sim }{F}$ be an approximate operator on *B* for a map *F* satisfying condition (13). Suppose $\{\ell _n\}$ generated by (15) for *F* and $\{\overset{\sim }{\ell _n}\}$ is defined as

28 $$\begin{aligned} {\left\{ \begin{array}{ll} \overset{\sim }{\ell }_1 = \overset{\sim }{\ell }\in B, \\ \overset{\sim }{\ell }_{n+1} = \overset{\sim }{F}((1-a_n) \overset{\sim }{u}_{n}+a_n \overset{\sim }{F}\overset{\sim }{u}_{n}), \\ \overset{\sim }{u}_n = \overset{\sim }{F}\overset{\sim }{v}_n , \\ \overset{\sim }{v}_n = \overset{\sim }{F}((1-b_n) \overset{\sim }{w}_{n}+b_n\overset{\sim }{F}\overset{\sim }{w}_{n}), \\ \overset{\sim }{w}_{n} = \overset{\sim }{F}((1-c_n) \overset{\sim }{\ell }_{n}+c_n \overset{\sim }{F}\overset{\sim }{\ell }_{n}), \end{array}\right. } \end{aligned}$$

with real sequences $\{a_n\}, \{b_n\}$ and $\{c_n\} \in [0,1]$ satisfying $\displaystyle \sum _{n=1}^{\infty } a_n =\infty$ and $\frac{1}{2} \le a_n$. If $F\varkappa = \varkappa$ and $\overset{\sim }{F}\overset{\sim }{\varkappa }= \overset{\sim }{\varkappa }$ such that $\displaystyle \lim _{n\rightarrow \infty }\overset{\sim }{\ell }_{n} = \overset{\sim }{\varkappa }$, then we have $\left| {\Vert {\varkappa -\overset{\sim }{\varkappa }} \Vert }\right| \le \dfrac{13\varepsilon }{1-\mu }$ where $\varepsilon$ is fixed.

#### Proof

Using (13), (15) and (28), we got,

$$\begin{aligned} \Vert {w_{n+1}-\overset{\sim }{w}_{n+1}} \Vert&=\Vert {F((1-c_n) \ell _{n}+c_n F\ell _{n})-\overset{\sim }{F}((1-c_n) \overset{\sim }{\ell }_{n}+c_n \overset{\sim }{F}\overset{\sim }{\ell }_{n})} \Vert \\&\preceq \Vert {F((1-c_n) \ell _{n}+c_n F\ell _{n})-F((1-c_n) \overset{\sim }{\ell }_{n}+c_n \overset{\sim }{F}\overset{\sim }{\ell }_{n})} \Vert \\ &+\Vert {F((1-c_n) \overset{\sim }{\ell }_{n}+c_n \overset{\sim }{F}\overset{\sim }{\ell }_{n})-\overset{\sim }{F}((1-c_n) \overset{\sim }{\ell }_{n}+c_n \overset{\sim }{F}\overset{\sim }{\ell }_{n})} \Vert \\&\preceq \mu (1-c_n) \Vert {\ell _{n}-\overset{\sim }{\ell }_{n}} \Vert +\mu c_n\Vert {F\ell _{n}-\overset{\sim }{F}\overset{\sim }{\ell }_{n}} \Vert +\varepsilon \\&\preceq \mu (1-c_n) \Vert {\ell _{n}-\overset{\sim }{\ell }_{n}} \Vert +\mu c_n\Vert {F\ell _{n}-F\overset{\sim }{\ell }_{n}+F\overset{\sim }{\ell }_{n}-\overset{\sim }{F}\overset{\sim }{\ell }_{n}} \Vert +\varepsilon \\&\preceq \mu (1-c_n) \Vert {\ell _{n}-\overset{\sim }{\ell }_{n}} \Vert +\mu ^2 c_n\Vert {\ell _{n}-\overset{\sim }{\ell }_{n}} \Vert +\mu c_n\varepsilon +\varepsilon \\&\preceq \mu (1-c_n+\mu c_n) \Vert {\ell _{n}-\overset{\sim }{\ell }_{n}} \Vert +\mu c_n\varepsilon +\varepsilon . \end{aligned}$$

$$\begin{aligned} \Vert {v_{n}-\overset{\sim }{v}_{n}} \Vert&=\Vert {F((1-b_n) w_{n}+b_n Fw_{n})-\overset{\sim }{F}((1-b_n) \overset{\sim }{w}_{n}+b_n \overset{\sim }{F}\overset{\sim }{w}_{n})} \Vert \\&\preceq \mu (1-b_n) \Vert {w_{n}-\overset{\sim }{w}_{n}} \Vert +\mu b_n\Vert {Fw_{n}-\overset{\sim }{F}\overset{\sim }{w}_{n}} \Vert +\varepsilon \\&\preceq \mu (1-b_n) \Vert {w_{n}-\overset{\sim }{w}_{n}} \Vert +\mu ^2 b_n\Vert {w_{n}-\overset{\sim }{w}_{n}} \Vert +\mu b_n\varepsilon +\varepsilon \\&\preceq \mu (1-b_n+\mu b_n) \Vert {w_{n}-\overset{\sim }{w}_{n}} \Vert +\mu b_n\varepsilon +\varepsilon . \end{aligned}$$

$$\begin{aligned} \Vert {u_{n}-\overset{\sim }{u}_{n}} \Vert&=\Vert {Fv_n-\overset{\sim }{F}\overset{\sim }{v}_n} \Vert \\&\preceq \Vert {Fv_n-F\overset{\sim }{v}_n} \Vert +\Vert {F\overset{\sim }{v}_n-\overset{\sim }{F}\overset{\sim }{v}_n} \Vert \\&\preceq \mu \Vert {v_n-\overset{\sim }{v}_n} \Vert +\varepsilon . \end{aligned}$$

Thus

$$\begin{aligned} \Vert {\ell _{n+1}-\overset{\sim }{\ell }_{n+1}} \Vert =&\Vert {F((1-a_n) u_{n}+a_n Fu_{n})-\overset{\sim }{F}((1-a_n) \overset{\sim }{F}\overset{\sim }{u}_{n}+a_n \overset{\sim }{F}\overset{\sim }{u}_{n}) } \Vert \\&\preceq \mu (1-a_n+\mu a_n) \Vert {u_{n}-\overset{\sim }{u}_{n}} \Vert +\mu a_n\varepsilon +\varepsilon \\&\preceq \mu (1-a_n+\mu a_n) \big (\mu \Vert {v_n-\overset{\sim }{v}_n} \Vert +\varepsilon \big )+\mu a_n\varepsilon +\varepsilon \\&= \mu ^2 (1-a_n+\mu a_n) \Vert {v_n-\overset{\sim }{v}_n} \Vert + \mu \varepsilon +\mu ^2 a_n \varepsilon +\varepsilon \\&\preceq \mu ^2 (1-a_n+\mu a_n) \big (\mu (1-b_n+\mu b_n) \Vert {w_{n}-\overset{\sim }{w}_{n}} \Vert +\mu b_n\varepsilon +\varepsilon \big ) + \mu \varepsilon +\mu ^2 a_n \varepsilon +\varepsilon \\&= \mu ^3 (1-a_n+\mu a_n) (1-b_n+\mu b_n) \Vert {w_{n}-\overset{\sim }{w}_{n}} \Vert +\mu ^3 b_n\varepsilon -a_n \mu ^3 b_n\varepsilon \\ &+\mu ^4 a_nb_n\varepsilon +\mu ^2\varepsilon +\mu ^3 \varepsilon a_n + \mu \varepsilon +\varepsilon \\&\preceq \mu ^3 (1-a_n+\mu a_n) (1-b_n+\mu b_n)\big (\mu (1-c_n+\mu c_n) \Vert {\ell _{n}-\overset{\sim }{\ell }_{n}} \Vert +\mu c_n\varepsilon +\varepsilon \big )\\&+\mu ^3 b_n\varepsilon -a_n \mu ^3 b_n\varepsilon +\mu ^4 a_nb_n\varepsilon +\mu ^2\varepsilon +\mu ^3 \varepsilon a_n + \mu \varepsilon +\varepsilon . \end{aligned}$$

29 $$\begin{aligned} \Vert {\ell _{n+1}-\overset{\sim }{\ell }_{n+1}} \Vert&\preceq \mu ^4 (1-a_n+\mu a_n) (1-b_n+\mu b_n) (1-c_n+\mu c_n) \Vert {\ell _{n}-\overset{\sim }{\ell }_{n}} \Vert +\mu ^4 c_n\varepsilon (1-a_n+\mu a_n) (1-b_n+\mu b_n) \nonumber \\ &+\mu ^3\varepsilon (1-a_n+\mu a_n) (1-b_n+\mu b_n)+\mu ^3 b_n\varepsilon +a_n \mu ^3 b_n\varepsilon (\mu -1)+\mu ^2\varepsilon +\mu ^3 \varepsilon a_n + \mu \varepsilon +\varepsilon . \end{aligned}$$

For $\mu \in (0, 1)$ and $a_n, b_n$ and $c_n$ are in [0, 1] $\forall n \in {\mathbb {N}}$, then we have the following observations $(1-a_n(1-\mu ))< 1, (1-b_n(1-\mu ))< 1, (1-c_n(1-\mu ))< 1, \mu , \mu ^2, \mu ^3, \mu ^4< 1, (\mu -1) < 0$ and $\mu ^3 a_n, \mu ^3 b_n, \mu ^3 c_n, \mu ^4 c_n < 1.$ Since our assumption that $\frac{1}{2}\le a_n$, we have $1-a_n \le a_n.$ Using the above observations together with (29), we get

30 $$\begin{aligned} \Vert {\ell _{n+1}-\overset{\sim }{\ell }_{n+1}} \Vert&\preceq (1-(1-\mu )a_n)\Vert {\ell _{n}-\overset{\sim }{\ell }_{n}} \Vert +a_n\varepsilon +6\varepsilon \nonumber \\&= (1-(1-\mu )a_n)\Vert {\ell _{n}-\overset{\sim }{\ell }_{n}} \Vert +a_n\varepsilon +6(1-a_n+a_n)\varepsilon \nonumber \\&\preceq (1-(1-\mu )a_n)\Vert {\ell _{n}-\overset{\sim }{\ell }_{n}} \Vert +a_n(1-\mu )\frac{13}{1-\mu }\varepsilon . \end{aligned}$$

Let $p_n = \Vert {\ell _{n}-\overset{\sim }{\ell }_{n}} \Vert , \delta _n = \frac{13}{1-\mu }\varepsilon , \nu _n = a_n(1-\mu )$. Using lemma (2.5) together with (30), we get

31 $$\begin{aligned} 0 \le \displaystyle \limsup _{n\rightarrow \infty }{p_n} \le \displaystyle \limsup _{n\rightarrow \infty }{\delta _n}. \end{aligned}$$

By Theorem (3.1), $\{\ell _n\}$ converges to $\varkappa$ of mapping *F* and the assumption that $\{\overset{\sim }{\ell _n}\}$ converges to a $\overset{\sim }{\varkappa }$ of mapping $\overset{\sim }{F},$ we obtain $\Vert {\varkappa -\overset{\sim }{\varkappa }} \Vert \le \dfrac{13\varepsilon }{1-\mu }$. $\square$

### Conclusion

In this work, we have proposed a new iterative process for approximating fixed points in complex-valued Banach spaces under contraction and weak contraction conditions. Through our analysis, we have demonstrated that our novel iterative approach achieves faster convergence rates compared to several existing methods, including the S-iterative, PMH, PKH, PIH, PSH, *HR*-iterative, and $HR^{*}$-iterative processes. Additionally, we have established the strong convergence of our new iterative process in complex-valued Banach spaces. Our findings are supported by both analytical proofs and numerical examples, and visualized the speed of convergence using MATLAB. Furthermore, we have extended our investigation to include a small result concerning non-expansive mapping using our proposed iterative technique. Finally, we have discussed the stability of our novel approach and its implications for data dependence under contraction conditions.

We can apply our fast iterative processes across various fields and industries. Some common areas where fast iterative algorithms find application include:

Optimization: Fast iterative algorithms are widely used in optimization problems across diverse domains such as engineering, finance, logistics, and machine learning. They help in finding optimal solutions to complex problems efficiently.

Signal Processing: In areas like image processing, audio signal processing, and communication systems, fast iterative algorithms are employed for tasks such as denoising, compression, filtering, and equalization.

Machine Learning and Data Mining: Iterative algorithms play a crucial role in training machine learning models, such as gradient descent-based optimization in neural networks, clustering algorithms like k-means, and dimensionality reduction techniques like principal component analysis (PCA).

Scientific Computing: Many scientific simulations and computations rely on fast iterative methods to solve differential equations, linear algebra problems, and optimization tasks arising from physics, chemistry, biology, and other scientific disciplines.

Finance and Economics: Iterative algorithms are used for portfolio optimization, risk management, option pricing, algorithmic trading, and other financial applications.

Computer Graphics and Vision: Fast iterative techniques are utilized in rendering algorithms, computer vision tasks like object detection and recognition, 3D reconstruction, and motion tracking.

Operations Research: In fields such as transportation, scheduling, and resource allocation, fast iterative methods are applied to solve complex optimization problems and improve decision-making processes.

Healthcare and Bioinformatics: Iterative algorithms find application in medical imaging, genome sequencing, drug discovery, and personalized medicine, aiding in data analysis, pattern recognition, and predictive modeling.

## Data Availability

The authors affirm that the data underpinning the results of this investigation are provided within the article itself.
